# Fair and Reasonable Allocation of Trans-Boundary Water Resources Based on an Asymmetric Nash Negotiation Model from the Satisfaction Perspective: A Case Study for the Lancang–Mekong River Bain

**DOI:** 10.3390/ijerph17207638

**Published:** 2020-10-20

**Authors:** Fang Li, Feng-ping Wu, Liu-xin Chen, Yue Zhao, Xiang-nan Chen, Zhi-ying Shao

**Affiliations:** 1 Business School, Hohai University, Nanjing 211100, China; lf@hhu.edu.cn (F.L.); jtsghrzm@yeah.net (L.-x.C.); zh_yyyyy@hhu.edu.cn (Y.Z.); 180213120002@hhu.edu.cn (X.-n.C.); shao_zy@hhu.edu.cn (Z.-y.S.); 2 National Engineering Research Center of Water Resources Efficient Utilization and Engineering Safety, Nanjing 210098, China

**Keywords:** trans-boundary water resources, multi-criteria decision making, flexible weight constraint, asymmetric Nash negotiation model, Lancang–Mekong River basin

## Abstract

The issue of trans-boundary water conflict has become an important factor affecting the relations between basin countries. The key to solve the current conflict problem is to realize the fair and reasonable allocation of trans-boundary water resources. Based on the satisfaction perspective, we developed an asymmetric Nash negotiation model to obtain an optimal and feasible allocation scheme for the trans-boundary water resources. Firstly, based on the two international water laws, we analyzed the influencing factors including water demand differences, resource endowment differences, and water efficiency differences, and by combing with the flexible weight constraint, we built the fair and reasonable allocation pattern for trans-boundary water resources. Secondly, under the constraint of the allocation pattern, we determined the ideal negotiation scheme of each basin country by considering their selection preference. Thirdly, we built a satisfaction degree function and established an asymmetric Nash negotiation model. This is used to build a fair negotiation mechanism among basin countries, and the allocation scheme after negotiation is regarded as the optimal allocation scheme. Lastly, we took the Lancang–Mekong river basin as an example. For this example, the results indicate the following: (1) after considering multiple factors comprehensively, China and Thailand obtained a higher proportion of trans-boundary water resource quota under different preference scenarios, while Myanmar obtained a lower proportion of trans-boundary water resource quota; (2) taking each basin country as the negotiation agent, the optimal allocation scheme with the introduction of fair negotiation mechanism has a higher degree of satisfaction, with an average of over 87.19%, which is more stable and easy to be accepted by all basin countries; (3) from the perspective of the change rate and the average satisfaction of the basin countries, the optimal allocation scheme under the resource endowment preference scenario obtained the highest satisfaction among basin countries. This study aims to improve the practicability and acceptability of trans-boundary water resources allocation, thus providing technical support for reducing trans-boundary water resources conflicts.

## 1. Introduction

Under the dual impact of global warming and high-intensity human activities, the problem of water shortage has become increasingly severe [1]. Trans-boundary river water resources are an important part of fresh water resources. There are 286 trans-boundary rivers globally involving 151 countries. About 42% of the global population live in trans-boundary river basins, and the annual runoff accounts for 54% of the global river flow [2]. With the aggravation of global water shortage, many countries have gradually begun to pay attention to the development and utilization of trans-boundary water resources. At present, more than 150 trans-boundary rivers may trigger international disputes. Unfair allocation and water resources utilization disputes have become the main causes of trans-boundary water security problems.

Conflicts in trans-boundary rivers have existed for a long time, posing a serious threat to the long-term peace and sustainable development of basin countries. In recent decades, to meet the various needs of different basin countries, some programmatic treaties have been produced internationally, including the “Helsinki Rules on the Uses of the Waters of International Rivers” [3] and “Convention on the Law of the Non-navigational Uses of International Watercourses” [4]. These are the most important legal documents in the process of adjusting the fair and reasonable utilization of international rivers. The two documents confirmed the criterion that should be considered in the process of trans-boundary water resources allocation, but the detailed indicators and the attribute weights are not determined. To date, a unified trans-boundary water resources allocation agreement has not been formed. Basin countries have widely different views or decisions in the allocation, development, utilization, and protection of trans-boundary water resources, because they often prioritize maximizing their own interests. Therefore, to reduce the conflicts caused by competitive use between basin countries, it is urgent to formulate a fair and reasonable allocation scheme.

How to achieve a fair and reasonable allocation of trans-boundary water resources has become the main focus and a difficult issue of current studies. At present, many research methods are available and widely used, including the following: (1) The index method, which has been widely used in the allocation of trans-boundary water resources, including the single index method and the multi-index method. Generally, the indicators involve basin population [5], water demand [6,7], and runoff contribution rate [8,9]. Fen and He [10] proposed that the trans-boundary water resource allocation should consider the specific hydrological characteristics, the socio-economic development level, and the actual water demand. Yu and Lu [11] analyzed the trans-boundary water resources allocation from the perspective of status quo, equity, and sustainable principles. (2) The optimization method, which focuses on describing the problem from the perspective of optimal objectives. Condon et al. [12] coupled a physics–hydrology–water resource management model and applied it to the water resource allocation of The Washita River in the United States. Guo [13] analyzed the multicriterion analysis and decision model for the trans-boundary water resources allocation. (3) The game method, which makes the allocation results closer to the real situation, and its application is more extensive. Based on the cooperative game model, Wang and Liu [14] proposed that the impact of external environment should be considered when allocating public river water resources. Safari et al. [15] discussed the interest coordination among water users by building a bargaining game model. Kucukmehmetoglu [16] proposed a water resources allocation method by integrating game theory and Pareto frontier concepts. Wu and Whittington [17] discussed the cooperation of water resources in the Nile basin and the coordination of interest relations among basin countries by applying the cooperative game model.

Using game theory to analyze trans-boundary water resource allocation is more practical than other methods [18], simulating the strategic choice of each agent. Nash negotiation theory, as a branch of game theory, was proposed by Nash in the 1950s as an effective method to solve the negotiation problem, i.e., participants solve the allocation problem through negotiation [19]. At present, some studies have applied this method for the income distribution and emission right distribution. Duan et al. [20] introduced the fair negotiation mechanism and obtained the optimization algorithm for total pollutant allocation. Duan and Li [21] discussed the income distribution of Public Privet Partnership (PPP) projects based on the asymmetric Nash negotiation model. As we know, in the allocation of trans-boundary water resources, there is no institution beyond national sovereignty; basin countries mainly reach relevant water allocation agreement through negotiation. Kampragou et al. [22] pointed out that there is a great risk of conflicts in the trans-boundary water resource allocation, and only through negotiation can conflicts be alleviated and water resource sharing be realized among countries. Wang et al. [23] believed that the initial allocation of trans-boundary water resources should be conducted by using the negotiation method.

To sum up, currently, studies have generally recognized the importance of trans-boundary water resource allocation. Although there are differences in research methods, studies all agree on the fairness and rationality of allocation schemes. From the research trend, we found the following: (1) More and more studies have changed from single index method to multi-index method. Moreover, various factors affecting the trans-boundary water resources allocation are analyzed and discussed by constructing a multicriterion decision-making (MCDM) model, improving the scientific and rationality of the allocation scheme. (2) Using game theory as a tool to analyze the trans-boundary water resources allocation is a relatively common and scientific method. Studies have been conducted on combing the optimization method and game theory method, providing scientific support for the fair and reasonable allocation of trans-boundary water resources.

However, some problems still need to be solved: firstly, the research on trans-boundary water resources allocation lags behind that in inland river basins, and specific allocation methods are still in its infancy; secondly, studies mainly focus on the formation of optimal allocation schemes, but few studies focused on whether the trans-boundary water resource allocation schemes can meet the reasonable demands of basin countries and be generally accepted by basin countries, and there is especially a lack of quantitative research.

The core issue of trans-boundary water resources allocation is what kind of standards and rules should be used to achieve the fair and reasonable allocation among basin countries. Moreover, the trans-boundary water resources allocation has the following characteristics: (1) fair and reasonable allocation of trans-boundary water resources should follow the provisions of international water laws; (2) trans-boundary water resources allocation is the optimal allocation scheme by considering the multi-factor and multi-standard comprehensively; (3) and basin countries can make joint decisions through negotiation to form the most satisfactory allocation scheme.

The Nash negotiation model can well simulate the negotiation process of each game subject and consider the individual reasonable interests. Based on the satisfaction perspective, this study developed an asymmetric Nash negotiation model by considering the multiple influencing factors, flexible weight constraint, and asymmetry power for the fair and reasonable allocation of trans-boundary water resources. The detailed steps are as follows: (1) Based on the two international water laws, an index system from the perspective of water demand differences, resource endowment differences, and water efficiency differences was designed. Considering the uncertainty of the weights of the indicators, a fair and reasonable allocation pattern was built by introducing the flexible weight constraints. The allocation pattern reflects the requirements of international law. This is also the basis for fair negotiation among basin countries. (2) Based on the constraints of allocation pattern, selection preferences of each basin country (BC) were considered according to their self-interest in order to determine the allocation scheme for negotiation of each BC. Since the sum of the ideal allocation ratio of each BC does not meet the constraint condition of equaling 100%, it enters into the stage of fair negotiation among the basin countries. (3) In the negotiation stage, an asymmetric Nash negotiation model is constructed using to obtain the optimal allocation scheme. This allocation scheme has a relatively high satisfaction degree through multiple rounds of negotiations.

**Figure 1:**
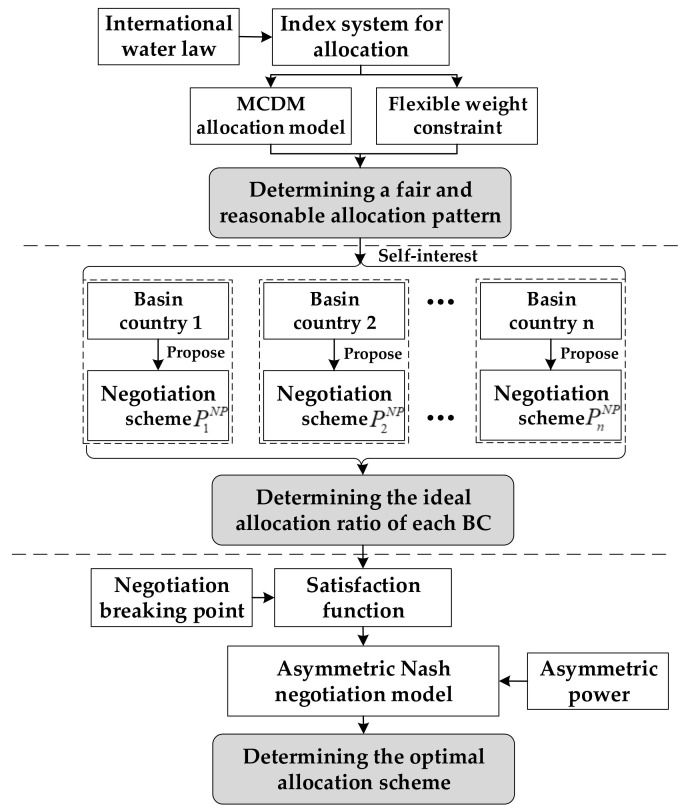
Logical structure of water resources allocation in trans-boundary river basins.

The rest of this study is organized as follows: In Section 2, the study area is introduced. In Section 3, the methods are introduced. In Section 4, the results are analyzed. In Section 5, the study is concluded. An outline is shown in Figure 1.

## 2. Case Study

**Figure 2:**
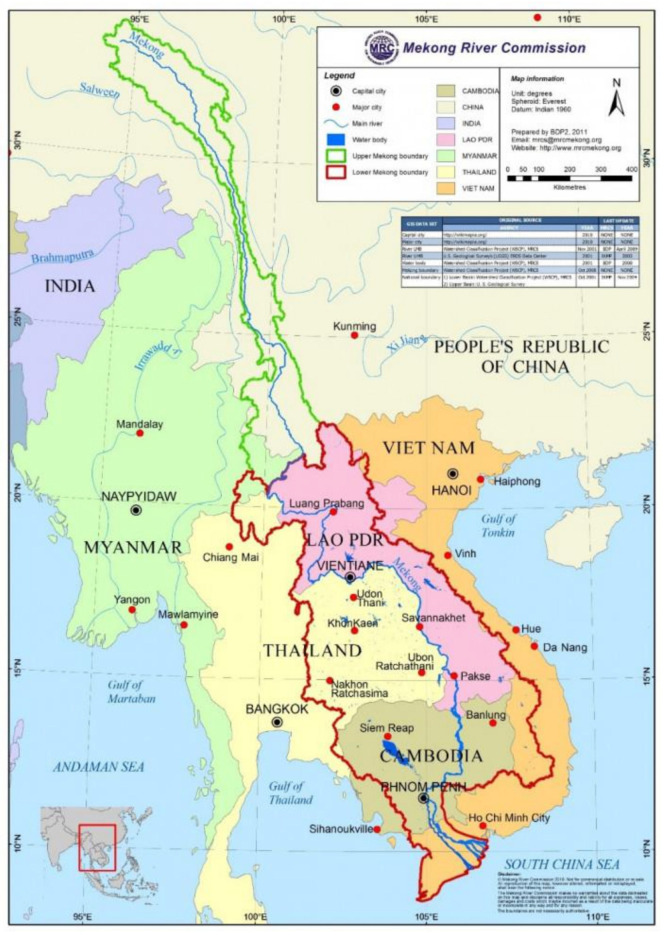
Geographical location of the LMRB [27].

The Lancang–Mekong River basin (LMRB) is one of the world largest rivers, originating from Qinghai, China, and entering Vietnam through Laos, Myanmar, Thailand, and Cambodia. It is a typical north–south river with complex and varied characteristics in its basin shape and distribution. The geographical location of the river basin is shown in Figure 2. The total length of LMRB is 4880 km. It is the sixth longest river in the world. The basin area is 81.0 × 10^4^ km, ranking the 12th over the world. The average flow is 10,560 $m^{3}/s$, and the total annual average runoff is 475 billion $m^{3}$. Most of the LMRB is in the tropical region, the annual rainfall distribution is unbalanced, and the river flow presents seasonal changes [24]. Therefore, although the LMRB is rich in total water resources, due to the influence of seasonal rainfall and basin distribution, the water demand and water supply do not match or even contradict with each other in the basin countries.

Due to the great differences in geographical location, industrial structure, energy demand composition, social economy, and culture among countries in the trans-boundary basins, there are many different interest demands for water resources development and utilization in the LMRB. China is located in the upstream, and the utilization of water resources is mainly based on hydropower generation. China has built dams and other infrastructure for its own development, arousing the concern of downstream countries about the decrease in available water, aggravating the water contradiction in the basin. Although Myanmar is in the upstream of LMRB, it occupies a small area and has a lower degree of development. Laos is located in the middle and lower reaches, and its main demand lies in accelerating the development of hydropower resources and supporting the agricultural water needs. However, its inappropriate development mode and uncontrolled use of water resources have led to ecological and environmental problems [25]. Thailand’s demand in the LMRB mainly includes agricultural irrigation water. Its farming schemes could lead to salinization of large areas and damage its ecological balance, causing dissatisfaction from downstream countries. Cambodia’s supporting industries are agriculture and fishing. To safeguard its own fishing development, Cambodia opposed upstream basin countries’ hydropower cascade development and diversion irrigation projects [26]. As the most downstream basin country, Vietnam has a large agricultural water demand and has a high consumption of water resources in the basin.

Based on their own development needs and the maximization of their own interests, basin countries are expected to obtain more water resources to support their own development. In addition, population increase, sustainable economic, and social development lead to the increasing demand for water resources in the LMRB, which also aggravate the water contradictions and conflicts among the basin countries. Therefore, it is urgent to make scientific and rational allocation of water resources in LMRB. This is the key problem to solve the water contradiction among basin countries and realize the sustainable utilization of water resources in the basin.

## 3. Methods

### 3.1. A Fair and Reasonable Allocation Pattern for Trans-Boundary Water Resources

#### 3.1.1. Establishment of an Index System

Due to the transnational and shared nature of trans-boundary water resources, it is necessary to construct an allocation standard and formulate a set of universally accepted allocation rules. In the existing studies and the practice of trans-boundary water resources allocation, there are mainly four different propositions, including demand theory, contribution theory, ability theory, and equal division theory. These four theories tend to use a single element for allocation, and although they are simple and convenient to operate, they are too one-sided and difficult to coordinate [13]. Trans-boundary water resources allocation should be the optimal allocation scheme considering the multi-factor and multi-standard comprehensively. The “Helsinki Rules on the Uses of the Waters of International Rivers” and “Convention on the Law of the Non-navigational Uses of International Watercourses” are used to adjust the fair and reasonable use of trans-boundary water resources. These two files listed the factors that need to be considered comprehensively, including the natural features of watershed, existing water use, and many other factors, providing the basis for the formulation of a fair, reasonable and acceptable trans-boundary water resources allocation scheme.

In this study, through comparative analysis of two international water laws and the reference of existing research, we analyzed the factors that influence the fair and reasonable allocation of trans-boundary water resources from the aspects of water demand differences, resource endowment differences and water efficiency differences. In the actual allocation negotiation, the basin countries can carry out in-depth joint research on the representational indexes and determine them through negotiation. In this study, by considering the representation, the availability, and practicability of the indexes, the index system was designed. The main influencing factors are as follows:

(1) Factors of water demand differences: By considering the current use and demand of water resources of each BC, the great influence on the current water use pattern in each BC can be avoided, improving the acceptability of the allocation scheme. The representational indexes including current water consumption (10^9^ m^3^), regional electricity demand proportion (%), population growth rate (%), and forest coverage rate (%).
(2) Factors of resource endowment differences: by considering the differences of resource endowment in population, area and water resources among the basin countries, we emphasized the contribution rate and dependence of each BC, which is fair and reasonable. The representational indexes including runoff contribution (m^3^/s), catchment area (10^4^ km^2^), river length (km), population (10^3^ people), and per capita water resource (m^3^/person). In this way, the natural equity and social equity of the allocation are guaranteed.
(3) Factors of water efficiency differences: by considering the water utilization efficiency of each BC, we thought that the proportion of water resources should be appropriately increased to the countries with higher water utilization efficiency in order to promote the development of water resource water-saving and improve water resource utilization technology within the region. The representational indexes including water productivity (USD/m^3^) and per capita GDP (USD/person).

Therefore, to sum up, the index system for fair and reasonable allocation of trans-boundary water resources is shown in Table 1.

#### 3.1.2. Multi-Criteria Decision Model for Allocation

Based on the above analysis, it was observed that the MCDM model is suitable for trans-boundary water resources allocation. The weighted synthesis method [29] was used to solve this problem. Set BC as $C_{i}\left(i=1,2,\cdots ,n\right)$, indicators as $f_{j}\left(j=1,2,\cdots ,m\right)$.The sample is set as $\left\{\left.v_{ij}\right|i=1,2,\cdots n;j=1,2,\cdots m\right\}$, where $v_{ij}$ indicates the attribute value of indicator $f_{j}$ of basin country $C_{i}$. Considering the dimensions of different indicators may be different, to facilitate the analysis, the normalized treatment method of linear transformation [31] was used to normalize the attribute values. Then, the normalized decision matrix $x_{ij}$ was obtained. The water demand differences composite index ($y_{1}$), endowment differences composite index ($y_{2}$), and the water efficiency differences composite index ($y_{3}$) of each BC were determined using the multi-index comprehensive evaluation method; the value of the composite index $y_{l,l=1,2,3}$ of each BC can be calculated as follows:

(1) $$\left\{\begin{array}{c} y_{i1}=\sum_{j=1}^{4}\alpha_{j}x_{ij}\\ y_{i2}=\sum_{j=5}^{8}\beta_{j}x_{ij}\\ y_{i3}=\sum_{j=9}^{11}\chi_{j}x_{ij} \end{array}\right.$$

where $\alpha_{j,j=1,2,\cdots ,4},\beta_{j,j=5,6,\cdots ,8},\chi_{j,j=9,10,11}$ is the weight of the indicator $f_{j}$.

To simplify the analysis, the analytic hierarchy process (AHP) method [22] was used to determine the weight of the detailed indicators. According to the composite index of water demand differences, endowment differences, and water efficiency differences of each BC, an allocation pattern was constructed based on a demand–endowment–efficiency difference composite index, as shown in Equation (2):

(2) $$R_{i}=\lambda_{1}\cdot y_{i1}+\lambda_{2}\cdot y_{i2}+\lambda_{3}\cdot y_{i3}$$

where $\lambda_{l,l=1,2,3}$ is the weight value of the composite index and satisfies $0<\lambda_{l,l=1,2,3}<1,\sum_{l=1}^{3}\lambda_{l}=1$.

Then the proportion of trans-boundary water resources in the basin countries can be determined as follows:

(3) $$p_{i}=R_{i}/\sum_{i=1}^{n}R_{i}$$

#### 3.1.3. Flexible Weight Constraint

It was observed from the above that the key to solve the allocation problem lies in determining the weight value of the composite index. International water laws listed the factors that should be considered in water resource allocation, but they do not determine the priority and weight of each factor. In the process of trans-boundary water resource allocation, each BC advocates the criterion of factors in its favor, making the problem of trans-boundary water resource allocation more complicated and more difficult to reach an agreement. Kampragou et al. [22] and Avarideh et al. [29] pointed out that lack of a unified and authoritative evaluation method for the weight determination of various factors, leading to contradictions and conflicts between basin countries. The basic agents of trans-boundary water resources allocation are the basin countries, and the determination of factor weights value should be determined by basin countries through negotiation under certain constraints. Therefore, referring to the interactive evaluation thought proposed by Wang [32], we set the composite index weight value as the flexible weight. Based on the actual demand, resource endowment, and water use efficiency of the country, each BC carries out negotiation under the flexible weight constrains. This can be expressed as Equation (4):

(4) $$\lambda =\left\{\lambda_{l}\left|\lambda_{l}\geq c;\lambda_{a}+\lambda_{b}\geq \lambda_{c},\forall l,a,b,c\in M;\sum_{l=1}^{3}\lambda_{l}=1\right.\right\}$$

where $\lambda_{l}\geq c$ is the equilibrium constraint, which means that the role of each factor in the water resources allocation should be balanced; $\lambda_{a}+\lambda_{b}\geq \lambda_{c},\forall a,b,c\in M$ is the non-dictatorial constraint; and $\sum_{l=1}^{3}\lambda_{l}=1$ is the general constraint and satisfies 0<c<1; $M=\left\{1,2,3\right\}$.

### 3.2. Determination of the Negotiation Scheme

Based on the fair and reasonable allocation pattern of trans-boundary water resources mentioned above, each BC will make a comprehensive consideration of their current situation, future demand, water resources endowment, and water use efficiency. Then, during negotiations, each BC puts forward its own ideal allocation scheme respectively, i.e., negotiation scheme (NS), and set $P_{i}^{NS}$ as the NS of $C_{i}$, as shown in Equation (5):

(5) $$P_{i}^{NS}=\left(p_{i1},p_{i2},\cdots ,p_{in}\right)$$

where $p_{ik}$ is the allocation proportion of basin country $C_{k}$ that is proposed by basin country $C_{i}$ and satisfies $0<p_{ik}<1,\sum_{k=1}^{n}p_{ik}=1$.

**Figure 3:**
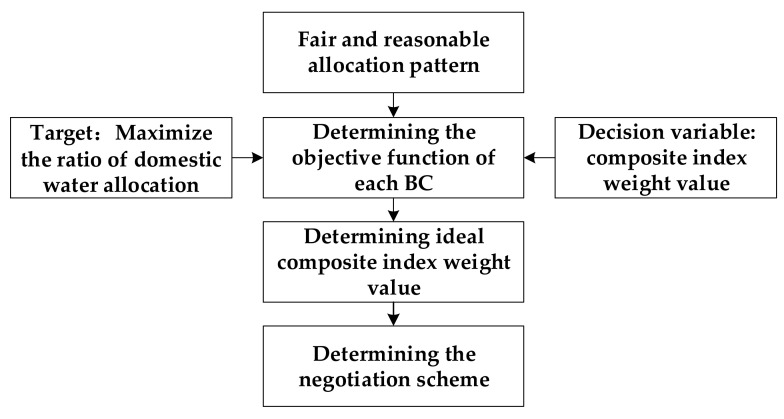
The process of determining the negotiation scheme of each BC.

The NS of each BC was determined by considering the selection preference of each BC. We assume each BC is a rational agent. When each BC negotiates with other basin countries, it tends to choose the weight value that is most conducive to its own water resources allocation [29,32]. Therefore, firstly, based on the idea of flexible weight, the composite index weight values $\lambda_{l,l=1,2,3}$ are set as the decision variable. Secondly, the objective function of each BC was constructed by referring to the fair and reasonable allocation pattern. Then, the ideal weight value and NS of each BC were obtained. The process of determining the negotiation scheme of each BC is shown in Figure 3.

According to Equation (3), the allocation ratio of $C_{i}$ was determined by domestic comprehensive evaluation value $R_{i}$ and other basin countries’ comprehensive evaluation value $R_{l}{{}_{,}}_{l\neq i}$. Therefore, according to the selection preference of $C_{i}$, set $\lambda_{il}$ represents the ideal weight value of the composite index $y_{l,l=1,2,3}$ of $C_{i}$. This is the decision variable, and the objective functions were determined by maximizing its own water resources allocation ratio, as shown in Equation (6):

(6) $$F_{i}=\max(R_{i}/\sum_{i=1}^{n}R_{i})$$

To facilitate the solution, we made a $\ln F_{i}$ and substituted in Equation (2). The objective function can be transformed as follows (See Appendix A):

(7) $$F_{i}^{\prime }=\max\left\{\ln(\sum_{l=1}^{3}\lambda_{il}\cdot y_{il})-\ln(\sum_{k,k\neq i}^{n}\left(\sum_{l=1}^{3}\lambda_{il}\cdot y_{kl}\right))\right\},i,k=1,2,\cdots ,n,\mathrm{and}i\neq k;l=1,2,3$$

Therefore, according to Equations (4) and (7), the objective function with constraints can be constructed using Equation (8):

(8) $$\begin{array}{l} F_{i}^{\prime }=\max\left\{\ln(\sum_{l=1}^{3}\lambda_{il}\cdot y_{il})-\ln(\sum_{k,k\neq i}^{n}\left(\sum_{l=1}^{3}\lambda_{il}\cdot y_{kl}\right))\right\}\\ s.t\left\{\begin{array}{l} \sum_{l=1}^{3}\lambda_{il}=1,i=1,2,\cdots ,n\\ \lambda_{il}\geq c,\forall i,l\\ \begin{array}{l} \lambda_{ia}+\lambda_{ib}\geq \lambda_{ic},\forall a,b,c\in \left\{1,2,3\right\}\\ 0<c<1 \end{array} \end{array}\right. \end{array}$$

Equation (8) was solved by using Lingo11.0. Then, the ideal weight value of the composite indexes $y_{l,l=1,2,3}$ that are proposed by $C_{i}$ can be obtained. After determining the ideal weight value $\lambda_{il}$, the ideal allocation scheme of each BC can be obtained by calculating Equations (2) and (3), set as $P_{i}^{NS}=\left(p_{i1},p_{i2},\cdots ,p_{ik},\cdots ,p_{in}\right)$, i.e., the negotiation scheme of $C_{i}$.

As is well known, the ideal allocation ratio for $C_{k}$ is $p_{k}^{+}=\underset{i=1\to n}{\max}\left\{p_{ik}\right\}$; the sum of the ideal allocation ratio cannot meet the constraint conditions that the sum of the allocation ratio should be 100%. Therefore, it entered into the stage of negotiation among the basin countries. They could not all satisfy their own benefits; therefore, they could only seek a compromise solution through negotiation. Thus, the allocation scheme is taken with the highest overall satisfaction as the optimal allocation scheme.

### 3.3. Asymmetric Nash Negotiation Model for the Trans-Boundary Water Resources

#### 3.3.1. Determination of the Asymmetry Power

Basin countries determine the allocation scheme through negotiation and consultation. To simplify the analysis, we do not consider the alliance relationship between basin countries, and we assume that there is no preference relationship among basin countries in the negotiation. In this process, there exits asymmetry among basin countries [33]. The economic, political, military and other differences inevitably affect the outcome of the negotiations [34]. In addition, the hydrological position of the BC is also an important resource power. Relevant studies have applied the asymmetric power (AP) to water resource allocation negotiations [34,35]. Therefore, referring to the AP considered in the existing studies, the AP index system from the perspectives of the hydrological position, economy, military, and politics was constructed. The value of AP was calculated using the projection method [36]. The detailed indexes are expressed in Table 2.

Hydrological location includes the geographical location of BC (-) with a range of 1–5. Generally, upstream countries can obtain more dominant power from geographical advantages [37]. This is a positive indicator.

Economic power includes per capita national income (current USD) and GDP growth (%). The higher the per capita national income, the richer the country [34] and the higher the bargaining power. GDP growth (%) is a positive indicator reflecting the economic development speed of the countries.

Military power includes the military force index (-) and the proportion of military expenditure (%). The military force index refers to the comprehensive index in the database of the world military observation organization [38], which is used to measure the military power of countries. The smaller the value, the stronger the military power of countries along the river basin. The proportion of military expenditure is expressed as the percentage of military expenditure in GDP, which reflects the importance attached by the basin countries to military expenditure.

Political influence include democracy level (score). In the long run, democracy will bring stability to a country, which can enhance a country’s position in the negotiations [35].

#### 3.3.2. Construction of the Asymmetric Nash Negotiation Model

By considering the complexity of trans-boundary water resources, it is difficult to determine its utility function accurately. In this study, the utility function of each BC was transformed into a linear function from the satisfaction perspective. For $C_{i}$, the greater the proportion of trans-boundary water resource quota, the higher the degree of satisfaction. The steps to determine the optimal allocation scheme are as follows:

**Step 1:** Determination of the ideal allocation ratio of each BC. Based on the above analysis, the ideal allocation ratio of $C_{k}$ can be expressed as $p_{k}^{+}=\underset{i=1\to n}{\max}\left\{p_{ik}\right\}$. Then, the allocation scheme which consisting of the ideal allocation ratio of each BC can be set as follows:

(9) $$P^{+}=\left(p_{1}^{+},p_{2}^{+},\cdots ,p_{k}^{+},\cdots ,p_{n}^{+}\right)$$

**Step 2:** Determination of the negotiation breaking point (NBP). Based on determining the ideal allocation ratio of each BC, it is observed that the sum of the ideal allocation ratio is greater than 100%, i.e., $\sum_{k=1}^{n}p_{k}^{+}>100\%$. To reach an agreed allocation scheme, basin countries need to make a certain degree of concessions, i.e., the basin countries need to bear a certain degree of water resources losses compared with their ideal allocation ratio. Set $p_{i}^{-}$ as the NBP of $C_{i}$, as shown in Equation (10):

(10) $$p_{i}^{-}=\left(1-\eta_{i}\right)p_{i}^{+}$$

where $\eta_{i}$ is the maximum acceptable concession parameter of $C_{i}$ and satisfies $0<\eta_{i}<1$. The bigger $\eta_{i}$ is, the more concessions the basin countries are willing to accept, and the cooperation is easier to obtain.

**Step 3:** Determination of the satisfaction function. Based on determining the ideal allocation ratio $p_{i}^{+}$ and the NBP $p_{i}^{-}$., the relationship between the optimal allocation ratio and the ideal allocation ratio can reflect the satisfaction degree of each BC to the optimal allocation scheme. From this, the utility function for each BC was defined [39]. Set the optimal allocation ratio after negotiation of $C_{i}$ as $p_{i}$. Firstly, the satisfaction degree of each BC was defined as follows:

(11) $$S_{i}=\frac{p_{i}}{p_{i}^{+}}$$

In addition, the lowest satisfaction degree of $C_{i}$ was defined as follows:

(12) $$S_{i}{}^{-}=\frac{p_{i}{}^{-}}{p_{i}^{+}}$$

**Step 4:** Establishment of the model. According to Equations (11) and (12), the asymmetric Nash negotiation model of each BC was constructed as follows:

(13) $$\begin{array}{l} Z^{\prime }=\max{\left(\frac{p_{1}}{p_{1}^{+}}-\frac{p_{1}{}^{-}}{p_{1}^{+}}\right)}^{\delta_{1}}\cdot {\left(\frac{p_{2}}{p_{2}^{+}}-\frac{p_{2}{}^{-}}{p_{2}^{+}}\right)}^{\delta_{2}}\cdots {\left(\frac{p_{n}}{p_{n}^{+}}-\frac{p_{n}{}^{-}}{p_{n}^{+}}\right)}^{\delta_{n}}\\ s.t\begin{array}{l} \left\{\begin{array}{l} p_{i}{}^{-}\leq p_{i}\leq p_{i}{}^{+}\\ \sum_{i=1}^{n}p_{i}=1 \end{array}\right. \end{array} \end{array}$$

The meanings of relevant parameters are the same as above. Using Lingo11.0 to solve Equation (13), the optimal allocation scheme of trans-boundary water resources with the highest overall satisfaction was obtained, i.e., $P^{optimal}=\left(p_{1},p_{2},\cdots ,p_{k},\cdots ,p_{n}\right)$

## 4. Results and Discussion

### 4.1. Scenarios and Data Sources

#### 4.1.1. Scenarios

Based on the above analysis, the fair and reasonable allocation of trans-boundary water resources was analyzed from the perspectives of water demand differences, resource endowment differences, and water efficiency differences. To further analyze the allocation results under different constrained scenarios, according to Equation (8), different constraint conditions according to the size of $\lambda_{1},\lambda_{2},\lambda_{3}$ were considered, including four scenarios: the general coupling scenario; demand preference scenario, resource endowment preference scenario, and efficiency preference scenario.

(1) Scenario 1: The general coupling scenario. In this scenario, the preference relationships of water demand, resource endowment, and water efficiency were determined by each BC according to the general constraint conditions of flexible weight, i.e., Equation (4).
(2) Scenario 2: The demand preference scenario. In this context, the actual water needs of basin countries were given priority, as shown in Equation (14):
  (14) $$\left\{\begin{array}{c} \lambda_{1}>\lambda_{2}\\ \lambda_{1}>\lambda_{3} \end{array}\right.$$
(3) Scenario 3: The resource endowment preference scenario. In this scenario, differences in resource endowment among basin countries were emphasized, as shown in Equation (15)
  (15) $$\left\{\begin{array}{c} \lambda_{2}>\lambda_{1}\\ \lambda_{2}>\lambda_{3} \end{array}\right.$$
(4) Scenario 4: The efficiency preference scenario. In this context, priority was given to the water efficiency of basin countries, as shown in Equation (16)
  (16) $$\left\{\begin{array}{c} \lambda_{3}>\lambda_{1}\\ \lambda_{3}>\lambda_{2} \end{array}\right.$$

#### 4.1.2. Data Sources

According to index system shown in Table 1, relevant data were collected through the Transboundary Freshwater Dispute Database (TFDD) [40], Transboundary Waters Assessment Programme (TWAP) database [2] and existing studies [19,41]. Among them, F_1_ is the sum of irrigation water consumption, industrial water consumption, and domestic water consumption. The details are shown in Table 3.

### 4.2. NS of Basin Countries in LMRB

Firstly, the data were processed by using the normalized method of linear transformation. Secondly, based on the AHP method, the weights of the detailed indicators were obtained. Then, the water demand differences composite index value ($y_{i1}$), endowment differences composite index value ($y_{i2}$), and water efficiency differences composite index value ($y_{i3}$) of each BC were calculated, as shown in Table 4.

According to Equation (8), without loss of generality, set c=5%. As mentioned in Section 4.1.1, by solving Equation (8), the NS of each BC that under the general coupling scenario can be obtained. To obtain other negotiation schemes of each BC under different preference scenarios, Equations (14)–(16) were combined with Equation (8), respectively. Then, the ideal composite index weight values $\lambda_{il}$ under different preference scenarios were obtained by using Lingo11.0. According to Equation (2) and Equation (3), the NS of each BC was determined and set as $P_{i}^{NS},i=1,2,\cdots ,6$, as shown in Table 5.

As Table 5 shows, in the four different scenarios, the NS that was mentioned by each BC cannot meet the constraint conditions that equal 100%, and because the trans-boundary water resources are shared by all basin countries, each BC cannot maximize their own benefits, and they have to make certain concessions for further cooperation.

### 4.3. AP of Basin Countries in LMRB

Based on the AP index shown in Table 2, specific index values are shown in Table 6.

The AP values of basin countries in LMRB were obtained using the projection method, as shown in Equation (17):

(17) $$\delta_{1}^{NP}=23.85\%,\delta_{2}^{NP}=5.63\%,\delta_{3}^{NP}=15.74\%,\delta_{4}^{NP}=17.33\%,\delta_{5}^{NP}=17.78\%,\delta_{6}^{NP}=19.68\%$$

### 4.4. Allocation Results in LMRB

According to Table 5, after determining the NS of each BC, that means, the ideal allocation ratio of each BC $p_{i}^{+}$ was determined. In addition, according to Equation (10), let $\eta_{i}=25\%,\forall i\in \left\{1,2,\cdots ,n\right\}$. Then, the NBP of each BC was calculated, as shown in Table 7.

By substituting the asymmetric power $\delta_{i}^{NP}$, ideal allocation ratio $p_{i}^{+}$, and negotiation breaking point $p_{i}^{-}$ into Equation (13), the optimal allocation scheme $P^{optimal}=\left(p_{1},p_{2},\cdots ,p_{k},\cdots ,p_{n}\right)$ under different preference scenarios was obtained, as shown in Table 8. The comparison results are as follows:

(1) According to the allocation ratio after negotiation, China and Thailand obtained higher allocation proportions of trans-boundary water resource quota under the four different allocation scenarios, while Myanmar obtained a lower proportion of trans-boundary water resource quota. The allocation quota of each BC under different scenarios was different. Among them, China obtained the highest allocation proportion under Scenario 4, which was 27.64%. This is mainly due to the low level of development and utilization of LMRB in China at the present stage, and the higher water use efficiency compared with other basin countries. In contrast Thailand obtained the highest allocation proportion under Scenario 2, which was 18.36%. This is mainly because Thailand had a large water demand at the present stage.
(2) According to the change rate, due to the small ideal allocation ratio that claims by Myanmar, its ideal allocation ratio has been satisfied completely. While in other basin countries, compared with their ideal allocation ratio, all of the optimal allocation ratios need to decrease with different degrees, ranging from −0.90% to −4.95%. The resource endowment preference scenario (Scenario 3) has a minimum range of variation, with an average range of −1.98%. Therefore, from the range of change before and after the negotiation, the allocation scheme under the resource endowment preference scenario is the least controversial scheme among the basin countries and is more easily accepted by them.

**Figure 4:**
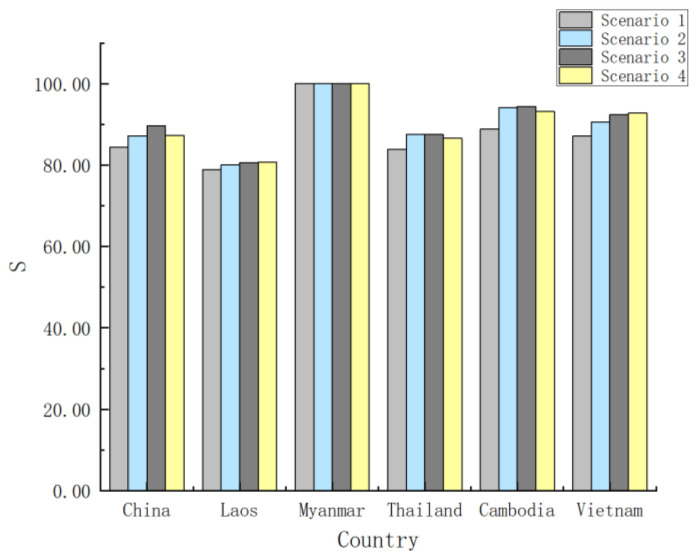
Satisfaction degree of each BC under different preference scenarios.

The satisfaction of each BC under different preference scenarios is shown in Figure 4. The comparison results are as follows:

(1) From the perspective of the satisfaction of each BC on the trans-boundary water resources allocation, satisfaction of each BC in the general coupling scenario is the lowest, indicating that the preference of different factors should be clarified in the specific allocation. As seen from Figure 4, different basin countries have different preferences. For example, China has the highest satisfaction under the scenario of resource endowment preference with a 89.61% degree of satisfaction, while Thailand has the highest satisfaction under the scenario of demand preference with a 87.54% degree of satisfaction.
(2) According to the average satisfaction of each BC on the allocation scheme, the average satisfaction under the four scenarios ranges from 87.19 to 90.73%. The average satisfaction under the scenario of resource endowment preference is the highest (90.73%). This is mainly because under the resource endowment preference scenario, more objective factors such as the contribution rate and the existing water resource endowment of each BC are considered, making it fairer and more reasonable. In the existing practice of trans-boundary water resources allocation, it also tends to give priority to the resource endowment differences of basin countries.

### 4.5. Stability Analysis of the Allocation Scheme in LMRB

To make more clear observation about the stability and acceptability of the allocation method that is proposed in this study, the compromise planning method proposed by Sun et al. [44] was used to construct the compromise power bankruptcy stability index (CPBSI), as shown in Equation (18):

(18) $$CPBSI=\sum_{i\in N}\delta_{i}^{NP}{\left(\frac{p_{i}-p_{i}^{+}}{p_{i}^{+}}\right)}^{2}$$

The meaning of specific parameters is the same as above. The smaller the CPBSI value, the more stable the result.

According to Equation (18), the stability of the NS ($P_{i}^{NS}$) and optimal allocation schemes $P^{optimal}$ of the basin countries under different preference scenarios can be calculated, as shown in Table 9.

According to the stability of different allocation schemes shown in Table 9, under the four scenarios, the CPBSI value of the optimal allocation scheme is the lowest, that is, the optimal allocation scheme has the highest stability. However, the CPBSI values of the negotiation schemes that are proposed by each BC are relatively high, indicating that the allocation of trans-boundary water resources cannot be decided by a certain BC, but should be decided by all the basin countries through negotiation based on certain rules. By constructing the agent satisfaction function, the optimal solution of trans-boundary water resources allocation with the consideration of all basin countries’ rational interests was obtained, improving the acceptability of the allocation scheme. In addition, in the resource endowment preference scenario, the CPBSI value is the lowest, i.e., 0.0091, which further indicates that trans-boundary water resources allocation scheme in the resource endowment preference scenario has the least controversy and is the most acceptable to all basin countries.

## 5. Conclusions

To reduce water conflict and realize the sustainable development of trans-boundary water resources, based on the asymmetric Nash negotiation game model, we simulated the consultative decision-making process among basin countries, and under the constraints of the international water laws, we introduced a negotiation mechanism to discuss the fair and reasonable allocation of trans-boundary water resources: (1) The differences of each BC including the water demand, resources endowment and water use efficiency were considered, and based on the flexible constraint weights, a fair and reasonable allocation pattern was built, improving the practicability of international water laws in trans-boundary water resources allocation. (2) With the BC as the negotiation agent, under the constraint of the fair and reasonable allocation pattern, each BC proposed its own NS based on the maximization of its own interests. By fully considering the reasonable requirements of each BC, the participation and initiative of each BC were enhanced. (3) From the perspective of geography, economics, and politics, the AP of each BC was determined, and the asymmetric Nash negotiation model was constructed to obtain an optimal allocation scheme, improving the acceptability of the trans-boundary water resources allocation scheme and effectively reducing trans-boundary water conflict.

We took LMRB as an example in this study. The results are as follows:

(1) The NS proposed by each BC based on the constraint of the fair and reasonable allocation pattern reflects the interest demands of each BC, but the sum of the ideal allocation ratios of each BC does not meet the constraint condition that equals 100%, so it is necessary for them to negotiate and undertake certain water resources losses. Moreover, the reduction range of allocation ratio undertaken by each BC in LMRB is between 0.00% and −4.95%.
(2) The average satisfaction degree of allocation schemes after negotiation optimization under different preference scenarios is more than 87.19%, which has a higher satisfaction and stability than the negotiation schemes proposed by each BC in LMRB. It shows that the optimal solution of trans-boundary water resources allocation based on negotiation can improve the feasibility and acceptability. From the perspective of the change rate before and after the negotiation, and the average satisfaction of the basin countries, the allocation scheme under the resource endowment preference scenario is better.
(3) The allocation scheme of LMRB is based on the utility function of each BC and considers the interest of each of them, subsequently enhancing the fairness and rationality of the allocation scheme, which provides a new idea for solving water conflicts.

Trans-boundary water resources allocation is complex work, and this study has some limitations: notably, the lack of an information sharing mechanism among the basin countries and the fact that basic data are difficult to obtain. In the future, effective cooperation among basin countries and sharing of information will be the key to carry out trans-boundary water resources allocation.

## Figures and Tables

**Table 1:** Index system of water resources allocation in trans-boundary River.

Target Level	The Index Type	Detailed Indicator	Label	Explanation
A fair and reasonable allocation of trans-boundary water resources	Water demand differences	Current water consumption (10^9^ m^3^)	F_1_	Describes the current utilization of trans-boundary water resources of each BC [28]. This is used to show respect for the current situation of water use. (+)
		Regional electricity demand proportion (%)	F_2_	Reflects the indirect demand of each BC for trans-boundary water resources. It measures the social and economic development demand [19]. (+)
		Population growth rate (%)	F_3_	Measures the changing trend of domestic water demand in each BC. (+)
		Forest coverage rate (%)	F_4_	Describes the ecological water needs of each BC. (+)
	Resource endowment differences	Runoff contribution (m^3^/s)	F_5_	Describes the hydrological conditions of each BC [8]. The greater the runoff contribution, the more water resources the BC should obtain. (+)
		Catchment area (10^4^ km^2^)	F_6_	Describes the geographical conditions of the BC and reflects the contribution of the BC. (+)
		River length (km)	F_7_	The greater the value, the more water resources the BC should obtain [29]. (+)
		Population (10^3^ people)	F_8_	If more people depend on trans-boundary water resources, more water resources should be allocated. (+)
		Per capita water resource (m^3^/person)	F_9_	Describes the domestic water resources situation in BC. This is used to describe other resources available to a BC and is a negative indicator. (-)
	Water efficiency differences	Water productivity (USD/m^3^)	F_10_	The higher the water productivity of the BC, the more GDP it can generate per unit of water, and the more water resources it should receive [19]. (+)
		per capita GDP (USD/person)	F_11_	This describes the economic and social development of the BC and is a positive indicator [30]. (+)

**Table 2:** Asymmetric powers in the negotiations among basin countries.

Index Type	Index	Label
Hydrological location	Geographical location of BC (-)	$H_{1}$
Economic power	Per capita national income (current USD)	$H_{2}$
	GDP growth (%)	$H_{3}$
Military power	Military force index (-)	$H_{4}$
	Proportion of military expenditure (%)	$H_{5}$
Political influence	Level of democracy (score)	$H_{6}$

**Table 3:** Index values of basin countries in the Lancang–Mekong River basin (LMRB).

Index	China	Laos	Myanmar	Thailand	Cambodia	Vietnam
F_1_	19.56	1.26	30.85	103.81	28.89	272.63
F_2_	70.31	0.4	0.98	15.76	0.25	12.3
F_3_	0.51	1.5	0.7	0.71	1.14	1.1
F_4_	55.7	81.3	44.5	32.1	53.6	47.6
F_5_	2410	5270	300	2560	2860	1660
F_6_	16.48	20.66	2.4	20.31	15.64	6.5
F_7_	2160	1987	265	976	502	230
F_8_	6710	6160	448	24,856	13,665	6904
F_9_	3450	31,151	21,071	3268	8626	4178
F_10_	14.9	2.8	2.3	6.7	6.8	1.8
F_11_	6807.43	1645.74	1152	5778.98	1007.57	1910.53

**Table 4:** Composite index value of each BC.

Value	China	Laos	Myanmar	Thailand	Cambodia	Vietnam
$y_{i1}$	0.4596	0.2305	0.1684	0.3448	0.2127	0.6625
$y_{i2}$	0.5564	0.6691	0.0725	0.7403	0.5188	0.3608
$y_{i3}$	1.0000	0.2059	0.1593	0.5828	0.3536	0.1741

**Table 5:** Negotiation scheme (NS) of each BC under different preference scenarios.

Scenarios	Country	$P_{1}^{NS}$	$P_{2}^{NS}$	$P_{3}^{NS}$	$P_{4}^{NS}$	$P_{5}^{NS}$	$P_{6}^{NS}$
Scenario 1: general coupling	China	31.68%	21.25%	30.78%	28.06%	28.06%	21.41%
	Laos	10.35%	17.81%	10.50%	16.39%	16.39%	17.23%
	Myanmar	6.86%	4.76%	6.94%	4.35%	4.35%	5.04%
	Thailand	20.85%	22.02%	20.51%	24.27%	24.27%	21.59%
	Cambodia	12.87%	14.80%	12.67%	16.03%	16.03%	14.44%
	Vietnam	17.39%	19.35%	18.60%	10.90%	10.90%	20.29%
Scenario 2: demand preference	China	31.23%	21.33%	30.78%	21.25%	21.33%	21.41%
	Laos	10.43%	17.52%	10.50%	17.81%	17.52%	17.23%
	Myanmar	6.90%	4.90%	6.94%	4.76%	4.90%	5.04%
	Thailand	20.68%	21.81%	20.51%	22.02%	21.81%	21.59%
	Cambodia	12.77%	14.62%	12.67%	14.80%	14.62%	14.44%
	Vietnam	17.99%	19.82%	18.60%	19.35%	19.82%	20.29%
Scenario 3: resource endowment preference	China	28.59%	21.25%	26.98%	28.06%	28.06%	21.33%
	Laos	16.02%	17.81%	14.79%	16.39%	16.39%	17.52%
	Myanmar	4.45%	4.76%	5.36%	4.35%	4.35%	4.90%
	Thailand	24.22%	22.02%	22.32%	24.27%	24.27%	21.81%
	Cambodia	15.94%	14.80%	14.52%	16.03%	16.03%	14.62%
	Vietnam	10.77%	19.35%	16.03%	10.90%	10.90%	19.82%
Scenario 4: efficiency preference	China	31.68%	28.59%	31.23%	28.59%	28.59%	28.59%
	Laos	10.35%	16.02%	10.43%	16.02%	16.02%	16.02%
	Myanmar	6.86%	4.45%	6.90%	4.45%	4.45%	4.45%
	Thailand	20.85%	24.22%	20.68%	24.22%	24.22%	24.22%
	Cambodia	12.87%	15.94%	12.77%	15.94%	15.94%	15.94%
	Vietnam	17.39%	10.77%	17.99%	10.77%	10.77%	10.77%

**Table 6:** Asymmetric power (AP) of basin countries in LMRB.

Index	China	Laos	Myanmar	Thailand	Cambodia	Vietnam
Geographical location of BC (-)	5	4	2	3	3	2
GDP per capita (USD/per) ^1^	10,261.68	2534.90	1407.81	7808.20	1643.12	2715.28
GDP growth (%) ^1^	6.11	4.65	2.89	2.37	7.05	7.02
Military force index (-) ^2^	0.0691	3.4433	0.5691	0.3571	2.0557	0.3559
Proportion of military expenditure (%) ^1^	1.87	0.19	2.92	1.33	2.21	2.30
Democracy level (score) ^3^	3.14	2.21	4.14	5.09	4.27	3.53

Data sources: ^1^ World Bank Database [42]; ^2^ Global Firepower Database [38]; ^3^ Economist Intelligence Unit (EIU) Database [43].

**Table 7:** Ideal allocation ratio and negotiation breaking point (NBP) of basin countries in LMRB.

Country	Ideal Allocation Ratio $\left(p_{i}^{+}\right)$				NBP $\left(p_{i}^{-}\right)$			
	Scenario 1	Scenario 2	Scenario 3	Scenario 4	Scenario 1	Scenario 2	Scenario 3	Scenario 4
China	31.68%	31.23%	28.59%	31.68%	23.76%	23.42%	21.45%	23.76%
Laos	17.81%	17.81%	17.81%	16.02%	13.36%	13.36%	13.36%	12.02%
Myanmar	6.94%	6.94%	5.36%	6.90%	5.20%	5.20%	4.02%	5.17%
Thailand	24.27%	22.02%	24.27%	24.22%	18.20%	16.51%	18.20%	18.17%
Cambodia	16.03%	14.80%	16.03%	15.94%	12.02%	11.10%	12.02%	11.96%
Vietnam	20.29%	20.29%	19.82%	17.99%	15.22%	15.22%	14.86%	13.49%

**Table 8:** Allocation ratio and changes of each BC in LMRB under different preference scenarios.

	Scenarios	China	Laos	Myanmar	Thailand	Cambodia	Vietnam
Allocation ratio after negotiation	Scenario 1	26.73%	14.06%	6.94%	20.36%	14.24%	17.67%
	Scenario 2	27.23%	14.26%	6.94%	19.28%	13.94%	18.36%
	Scenario 3	25.62%	14.35%	5.36%	21.23%	15.13%	18.30%
	Scenario 4	27.64%	12.94%	6.90%	20.99%	14.85%	16.69%
Compared with the ideal allocation ratio	Scenario 1	−4.95%	−3.75%	0.00%	−3.91%	−1.80%	−2.62%
	Scenario 2	−4.00%	−3.56%	0.00%	−2.74%	−0.87%	−1.93%
	Scenario 3	−2.97%	−3.47%	0.00%	−3.03%	−0.90%	−1.51%
	Scenario 4	−4.04%	−3.09%	0.00%	−3.23%	−1.09%	−1.30%

**Table 9:** Stability analysis of different allocation schemes under different scenarios in LMRB.

	Scenarios	$P_{1}^{NS}$	$P_{2}^{NS}$	$P_{3}^{NS}$	$P_{4}^{NS}$	$P_{5}^{NS}$	$P_{6}^{NS}$	$P^{optimal}$
CPBSI	Scenario 1	0.0243	0.0442	0.0230	0.0675	0.0675	0.0408	0.0183
	Scenario 2	0.0162	0.0377	0.0154	0.0402	0.0377	0.0356	0.0112
	Scenario 3	0.0461	0.0203	0.0123	0.0458	0.0458	0.0197	0.0091
	Scenario 4	0.0172	0.0538	0.0176	0.0538	0.0538	0.0538	0.0109

## Abbreviations

PPP	Public Privet Partnership
MCDM	Multi-criteria decision model
BC	Basin country
LMRB	Lancang–Mekong River Bain
AHP	Analytic hierarchy process
NS	Negotiation scheme
AP	Asymmetric power
NBP	Negotiation breaking point
CPBSI	Compromise power bankruptcy stability index

## Appendix A

According to Equation (6), we can derive

(A1) $$F_{i}=\max\left(R_{i}/\sum_{i=1}^{n}R_{i}\right)=\max\left\{R_{i}/\left(R_{i}+\sum_{k\neq i}^{n}R_{k}\right)\right\}=\max\left\{1/\left(1+\sum_{k\neq i}^{n}R_{k}/R_{i}\right)\right\}$$

To solve (A1), equivalently to solve $F_{i}^{\prime }$ as:

(A2) $$F_{i}^{\prime }=\min\left(\sum_{k\neq i}^{n}R_{k}/R_{i}\right)$$

To solve (A2), equivalently to solve $F_{i}^{\prime \prime }$:

(A3) $$F_{i}^{\prime \prime }=\max\left(R_{i}/\sum_{k,k\neq i}^{n}R_{k}\right)$$

Then, we make Ln ($F_{i}$) of (A3), and substituted in Equation (2). It can be explained as:

(A4) $$F_{i}^{\prime \prime }=\max\left\{\ln\left(\sum_{l=1}^{3}\lambda_{il}\cdot y_{il}\right)-\ln\left(\sum_{k\neq i}^{n}\left(\sum_{l=1}^{3}\lambda_{kl}\cdot y_{kl}\right)\right)\right\},i,k=1,2,\cdots ,n,and\;i\neq k;l=1,2,3$$

## References

[1] Li F., Wu F.P., Chen L.X., Xu X. (2020). Hawk and dove game model of trans-boundary river conflict and cooperation under asymmetric perspective. China Popul. Resour. Environ..

[2] Transboundary Waters Assessment Programmer (TWAP) Database. http://twap-rivers.org/#home.

[3] International Law Association (ILA) (1996). Helsinki Rules on the Uses of the Waters of International Rivers. Report of the Fifty-Second Conference of the International Law Association.

[4] UN Watercourses Convention (1997). Convention on the Law of the Non-Navigational Uses of International Watercourses.

[5] Feng Y., He D., Li Y. (2013). The key indicators of transboundary water apportionment based on international laws and cases. J. Geogr. Sci..

[6] Gorgoglione A., Crisci M., Kayser R.H., Chreties C., Collischonn W. (2019). A New Scenario-Based Framework for Conflict Resolution in Water Allocation in Trans-boundary Watersheds. Water.

[7] Liu Y.L., Zhao Z.X., Sun Z.L., Wang G.Q., Jin J.L., Wang G.X., Bao Z.X., Liu C.S., He R.M. (2019). Multi-objective water resources allocation in trans-boundary Rivers based on the concept of water benefit-sharing: A case in the Lancang-Mekong River. Scientia Geographica Sinica.

[8] Wang Z.J. (2017). Applicability of the Principle of Reciprocity of Rights and Obligations in International River Water Utilization—and Construction of International River Water Rights System.

[9] Basheer M., Wheeler K.G., Ribbe L., Majdalawi M., Abdo G., Zagona E.A. (2018). Quantifying and evaluating the impacts of cooperation in transboundary river basins on the Water-Energy-Food nexus: The Blue Nile Basin. Sci. Total. Environ..

[10] Fen Y., He D.M. (2003). Study on water right, available utilization and protection of water resources in international rivers. Adv. Water Sci..

[11] Yu S., Lu H.W. (2018). An integrated model of water resources optimization allocation based on projection pursuit model–Grey wolf optimization method in a transboundary river basin. J. Hydrol..

[12] Condon L.E., Maxwell R.M. (2013). Implementation of a linear optimization water allocation algorithm into a fully integrated physical hydrology model. Adv. Water Resour..

[13] Guo S.Z. (2014). Theoretical and Empirical Research on the Construction of the System of International River Water Rights. Ph.D. Thesis.

[14] Wang X.J., Liu J. (2020). A cooperative game model for international water sharing problems. Chin. J. Manag. Sci..

[15] Safari N., Zarghami M., Szidarovszky F. (2014). Nash bargaining and leader–follower models in water allocation: Application to the Zarrinehrud River basin, Iran. Appl. Math. Model..

[16] Kucukmehmetoglu M. (2012). An integrative case study approach between game theory and Pareto frontier concepts for the transboundary water resources allocations. J. Hydrol..

[17] Wu X., Whittington D. (2006). Incentive compatibility and conflict resolution in international river basins: A case study of the Nile Basin. Water Resour. Res..

[18] Zhang Z., He W., An M., Degefu D.M., Juqin S., Yuan L. (2019). Multi Game Theory Analysis of Cooperation Stability of Trans-boundary Water Pollution Governance. Nat. Environ. Pollut. Technol..

[19] Yuan L., Shen J.Q., He W.J., Degefu D.M. (2018). Bankruptcy game of transboundary river water resources allocation based on subject inequality. J. Hohai Univ. Philos. Soc. Sci..

[20] Duan H.Y., Wang P.B., Cai F.F., Zhao J.C., Wang X.E. (2018). Difference fairness allocation and the optimization algorithm of the provincial total amount control index of pollutant emissions: Based on asymmetric Nash negotiation model. China Popul. Resour. Environ..

[21] Duan S.X., Li T. (2019). Research on profit distribution of PPP project based on asymmetrical Nash negotiation model. J. Ind. Technol. Econ..

[22] Kampragou E., Eleftheriadou E., Mylopoulos Y. (2007). Implementing Equitable Water Allocation in Transboundary Catchments: The Case of River Nestos/Mesta. Water Resour. Manag..

[23] Wang L.Z., Fang L., Hipel K.W. (2003). Water Resources Allocation: A Cooperative Game Theoretic Approach. J. Environ. Inform..

[24] Mekong River Commission (2018). Annual Mekong Hydrology, Flood and Drought Report.

[25] Sun Z.L., Liu Y.L., Liu J., Zhao Z.X., Wang G.X., Jin J.L., Bao Z.X., Liu C.S. (2018). Analysis on the present situation and demand of water utilization in the Lancang-Mekong River Basin. J. Water Resour. Water Eng..

[26] Wen Y.D. (2016). A Study on the Allocation of the Water Resources of Lancang-Mekong River. Ph.D. Thesis.

[27] Mekong River Commission. http://www.mrcmekong.org/news-and-events/events/mrc-secretariat-affirms-mekong-basin-size-length/.

[28] Zeng Y., Li J., Cai Y., Tan Q. (2017). Equitable and reasonable freshwater allocation based on a multi-criteria decision-making approach with hydro-logically constrained bankruptcy rules. Ecol. Indic..

[29] Avarideh F., Attari J., Moridi A. (2017). Modelling Equitable and Reasonable Water Sharing in Transboundary Rivers: The Case of Sirwan-Diyala River. Water Resour. Manag..

[30] Li B., Zhang K., Cheng T.J. (2018). Application of AHP to Lancang River-Mekong River consumable water distribution. Eng. J. Wuhan Univ..

[31] Wu F.P., Chen Y.P. (2011). Modern Decision Making Method.

[32] Wang J.F. (2014). Competitive decision method of the initial emission rights allocation under total quantity control. China Popul. Resour. Environ..

[33] Ansink E., Weikard H.P. (2009). Contested water rights. Eur. J. Political Econ..

[34] Qin J., Fu X., Peng S., Xu Y., Huang J., Huang S. (2019). Asymmetric Bargaining Model for Water Resource Allocation over Transboundary Rivers. Int. J. Environ. Res. Public Health.

[35] Sheikhmohammady M., Hipel K.W., Kilgour D.M. (2012). Formal analysis of multilateral negotiations over the legal status of the Caspian Sea. Group Decis. Negot..

[36] Lu W.F., Tan H.X. (2012). A comprehensive improvement on the TOPSIS method for multi-attribute decision making. Stat. Decis..

[37] Li X.L. (2016). Conflict or cooperation: Paths and mechanisms of transnational river water management. Foreign Aff. Rev..

[38] Global Firepower (GFP) Database. https://www.globalfirepower.com/.

[39] Read L., Madani K., Inanloo B. (2014). Optimality versus stability in water resource allocation. J. Environ. Manag..

[40] Transboundary Freshwater Dispute Database (TFDD). https://transboundarywaters.science.oregonstate.edu/content/about-us/.

[41] Zhao Z.X., Liu Y.L., Wang Y.N., Deng X.Y., Yan B., Gen L.H. (2018). Construction of multi-level ownership system of cross-border water resources—taking Lancang-Mekong river basin as an example. Water Resour. Dev. Res..

[42] World Bank. https://data.worldbank.org.cn/country.

[43] Economist Intelligence Unit (EIU) Database. http://www.eiu.com/home.aspx#.

[44] Sun D.Y., Wang H.M., Chu Y. (2015). Application of bankruptcy theory in resolving water resource allocation conflict over trans-boundary rivers. China Popul. Resour. Environ..

